# Hepatoprotective Effects of *Orthosiphon stamineus* Extract on Thioacetamide-Induced Liver Cirrhosis in Rats

**DOI:** 10.1155/2011/103039

**Published:** 2011-05-05

**Authors:** Mohammed A. Alshawsh, Mahmood Ameen Abdulla, Salmah Ismail, Zahra A. Amin

**Affiliations:** Department of Molecular Medicine, Faculty of Medicine, University of Malaya, Kuala Lumpur 50603, Malaysia

## Abstract

*Orthosiphon stamineus* as medicinal plant is commonly used in Malaysia for treatment of hepatitis and jaundice; in this study, the ethanol extracts were applied to evaluate the hepatoprotective effects in a thioacetamide-induced hepatotoxic model in *Sprague Dawley* rats. Five groups of adult rats were arranged as follows: Group 1 (normal control group), Group 2 Thioacetamide (TAA) as positive control (hepatotoxic group), Group 3 Silymarin as a well-known standard drug (hepatoprotective group), and Groups 4 and 5 as high and low dose (treatment groups). After 60-day treatment, all rats were sacrificed. The hepatotoxic group showed a coarse granulation on the liver surface when compared to the smooth aspect observed on the liver surface of the other groups. Histopathological study confirmed the result; moreover, there was a significant increase in serum liver biochemical parameters (ALT, AST, ALP, and Bilirubin) and the level of liver Malondialdehyde (MDA), accompanied by a significant decrease in the level of total protein and Albumin in the TAA control group when compared with that of the normal group. The high-dose treatment group (200 mg/kg) significantly restored the elevated liver function enzymes near to normal. This study revealed that 200 mg/kg extracts of *O. stamineus* exerted a hepatoprotective effect.

## 1. Introduction

The liver is an important organ responsible for the metabolism, bile secretion, elimination of many substances, blood detoxifications, synthesizes, and regulation of essential hormones. Liver diseases have become a worldwide problem and are associated with significant morbidity and mortality. The principal causative factors for the liver diseases in developed countries are excessive alcohol consumption, and viral-induced chronic liver diseases while in the developing countries the most frequent causes are environmental toxins, parasitic disease, hepatitis B and C viruses, and hepatotoxic drugs (certain antibiotics, chemotherapeutic agents, high doses of paracetamol, carbon tetrachloride (CCL_4_), thioacetamide (TAA), etc.). Chronic liver cirrhosis and drug induced liver injury accounting the ninth leading cause of death in western and developing countries [1]. In the absence of reliable hepatoprotective drugs in modern medicine, a large number of herbal preparations have become increasingly popular for the treatment of liver disorders [2]. A number of herbals show promising activity, including Silymarin for liver cirrhosis, *Phyllantus amarus* in chronic hepatitis B, glycyrrhizin to treat chronic viral hepatitis, and some herbal combinations from China and Japan that have been scientifically proven for treatment of liver diseases [3]. Silymarin, a flavonolignan from “milk thistle” *Silybum marianum, *is widely used for hepatoprotection. Silymarin offers good protection in different toxic models of induced liver cirrhosis experiments by using laboratory animals. 

*Orthosiphon stamineus *Benth (Family: Lamiaceae), named Misai kucing (Malaysia), kumis kucing (Indonesia), and Java tea (Europe), this is native plant to South East Asia [4]. *O. stamineus *has been widely used in Malaysia for treating kidney problems, fever, hypertension, gout, diabetes, hepatitis, and jaundice [5, 6]. The literature review shows that this plant contains phenolic compounds and flavonoids. More than twenty phenolic compounds were isolated from *O. stamineus*, the most important constituents are nine lipophilic flavones, two flavonol glycosides, and nine caffeic acid derivatives [7]. The well-known chemical constituents of *O. stamineus *are caffeic acid, cirrchoric acid, diterpenes, orthosiphols, monoterpenes, triterpenes, saponins, hexoses, organic acids, rosmarinic acids, sinensetin, eupatorin, and 3′-hydroxyl-5,6,7,4′-tetramethoxyflavone [8–10]. *O. stamineus *has been proven using animal models to treat diabetes mellitus and improving lipid profile in diabetic rats [11], kidney problem diuretic and hypouricemic effects in rats [12], as anti-inflammatory [13], for the treatment of hypertension [14], and antipyretic activity [15]. The experimental induction of liver cirrhosis by long exposure of Thioacetamide results in histological and biochemical changes similar to that of human liver cirrhosis [16]. The TAA model is more reliable and easy for induced liver cirrhosis than the CCl_4_ model [17]. This study was carried out to assess the hepatoprotective activity of *O. stamineus *against thioacetamide-induced hepatotoxicity in rats to prove scientifically the traditional use of this plant against liver disorders.

## 2. Materials and Methods

### 2.1. Plant Materials and Chemicals

*O. stamineus *plant leaves were obtained from the Ethno Resource Sdn Bhd. The plant was identified, and voucher specimen was kept in our laboratory for future references. The dried and powdered leaves (100 gm) were extracted with 900 mL of 95% ethanol for 48 hour, and the ethanol extract was filtered and evaporated under low pressure by using Buchi-type rotary evaporator to give the crude-dried extract. The percentage yield of ethanol extracts was found to be 8.1% (w/w). The dry extract was then dissolved in Tween 20 (10% w/v) and administered orally to rats in concentrations of 100 and 200 mg/kg body weight.

Thioacetamide from (Sigma-Aldrich, Switzerland) and all other chemicals used were of analytical grade and purchased mostly from Sigma-Aldrich and Fisher. The chemical was dissolved in sterile distilled water and injected intraperitoneally to the rats in concentrations of 200 mg/kg body weight [18]. Silymarin (International Laboratory, USA) as a standard drug and was dissolved in Tween 20 (10% w/v) and orally administered to rats in concentrations of 50 mg/kg body weight [19].

### 2.2. Total Phenolic and Flavonoids Determination

The *O. stamineus* extract was evaluated for their total phenolic content by using Folin-Ciocalteu reagent and was calculated as gallic acid equivalents in mg (GAE)/g of extract according to Folin-Denis colorimetric method [20]. However, the total flavonoids was determined by using the aluminium chloride colorimetric method and expressed as quercetin equivalents in mg (QE)/g of extract as described by Dowd [21]. Both assays were carried out in triplicate.

### 2.3. Animals

Adult male healthy *Sprague Dawley* (SD) rats weighing 200–250 gm were obtained from Animal House Unit, Faculty of Medicine, University of Malaya, Malaysia. They were kept in wire-bottomed cages at 25 ± 3°C temperature, 50–60% humidity, and a 12 h light-dark cycle for at least a week before the experiment. They were maintained at standard housing conditions and free access to standard diet and water ad libitum during the experiment. The experimental protocol was approved by Animal Ethics Committee; with an ethic no. (PM 28/08/2009/MAA (R). Throughout the experiments, all criteria of taking care of animals prepared by the National Academy of Sciences and outlined in the “Guide for the Care and Use of laboratory Animals” were applied.

### 2.4. Experimental Design

The animals were randomly divided into five groups of eight rats each and treated as follows.

**Group 1**

10% Tween 20 (5 mL/kg, po) daily for 2 months + sterile distilled water (1 mL/kg, i.p) thrice weekly for 2 months (normal control group).

**Group 2**

10% Tween 20 (5 mL/kg, po) daily for 2 months + TAA (200 mg/kg, i.p) thrice weekly for 2 months (positive control hepatotoxic group).

**Group 3**

Silymarin (50 mg/kg, po) daily for 2 months + TAA (200 mg/kg, i.p) thrice weekly for 2 months (well known standard drug hepatoprotective group).

**Group 4**

*O. stamineus* (200 mg/kg, po) daily for 2 months + TAA (200 mg/kg, i.p) thrice weekly for 2 months (treatment group, high dose).

**Group 5**

*O. stamineus* (100 mg/kg, po) daily for 2 months + TAA (200 mg/kg, i.p) thrice weekly for 2 months (treatment group, low dose).

Body weights of all animals were measured every week. All rats were sacrificed 24 hours after last treatment and overnight fasting under diethyl ether anesthesia. Blood samples were collected; serum was separated for assay of the liver biomarker. The liver and spleen were harvested, washed in normal saline, blotted with filter paper, and weighed. Gross examination was conducted to examine of any abnormalities developed in the organs. The liver of all animals was subsequently subjected to histopathological examination in a blinded fashion.

### 2.5. Biochemical and Histopathological Examination

The collected blood samples were separated at 2500 rpm for 15 minutes after been completely become clotted. Serum for assay of the liver biomarkers such as Aspartate aminotransferase (AST), Alanine aminotransferase (ALT), Alkaline phosphatase (ALP), Bilirubin, Total protein (TP), and Albumin was assayed spectrophotometrically by standard automated techniques according to the procedures described by the manufacturers in Central Diagnostic Laboratory, University of Malaya Medical Centre. The Liver was sliced and pieces were fixed in 10% buffered formaldehyde solution for histological study. The fixed tissues were processed by automated tissue processing machine. Tissues were embedded in paraffin wax by conventional methods. Sections of 5 *μ*m in thickness were prepared and then stained with hematoxylin-eosin (HE). After that the sections were observed under the microscope for histopathological changes, and their photomicrographs were captured. 

### 2.6. Estimation of Malondialdehyde (MDA) in Liver Tissue

Liver samples were washed immediately with ice-cold saline to remove as much blood as possible. Liver homogenates (10% w/v) were prepared in a cold 50 mM potassium phosphate buffer (pH 7.4) using homogenizer in ice. The cell debris was removed by centrifugation at 4500 rpm for 15 at 4°C using refrigerated centrifuge. The supernatant was used for the estimation of Malondialdehyde (MDA) level by using (Cayman Chemical Company, U.S.A) kit.

### 2.7. Statistical Analysis

The statistical significance was assessed using one-way analysis of variance (ANOVA) followed by Bonferroni's multiple comparison test. All values were expressed as mean ± S.E.M., and a value of *P* < .05 was considered significant as compared to the respective control group using SPSS programme for windows version 18 (SPSS Inc. Chicago, IL, USA).

## 3. Results

### 3.1. Body, Liver, and Spleen Weight

Before the treatment was started the rats weighed 200–250 g and after two months animals of normal, HD, LD, and Silymarin groups reached average body weights of 254.9, 232.7, 263.3, and 257.0 g, respectively. However, TAA positive control group the average body weight was decreased to 202.0 g but without a significant difference compared to the normal control group. There was no significant difference between the groups but long-term taken of TAA led to significant increase of the liver weight compared to normal rats. Values of mean relative liver weight (LW/BW) percent showed a significant difference between treated groups compared to TAA group (Table 1). 

### 3.2. Biochemical and Antioxidant Parameters

Long-term taken of TAA led to significant increase of biochemical markers ALT, AST, ALP, Bilirubin, and MDA level, while significantly decreased total protein and albumin compared to the normal control group, indicating acute hepatocytes damage. Treatment of animals with *O. stamineus* extracts and Silymarin significantly reduced the level of liver function biomarker (ALT, AST, ALP, and bilirubin) and antioxidant parameter (MDA), in addition significantly increased in total protein and albumin comparing with the thioacetamide group. The toxic effect of TAA was controlled in the rats treated with ethanolic extracts (100 mg/kg and 200 mg/kg) and that is approved by restored of the levels of the liver biomarker. At a dose of 100 mg/kg, the effect was only marginal, whereas at the higher dose (200 mg/kg) the extract effectively prevented the TAA-induced liver damage (Table 2). The ethanol extracts of *O. stamineus* significantly restored the altered liver parameters and made it more resemble to that of standard drug Silymarin (50 mg/kg). Moreover, *O. stamineus *extract at 200 mg/kg (*P* < .05) demonstrated the most potent effect in protecting rats against TAA-induced liver damage, as evidenced by the reduced in all enzyme levels of AST, ALT, and ALP and increased in total protein and albumin levels compared to the control. On the other hand, the total phenolic contents were 294.3 ± 0.005 mg (Gallic acid equivalents) per g of extracts (standard curve equation: *y* = 0.0013*x* + 0.0032, *R* ^²^ = 0.987). At the same time, flavonoids were 171.4 ± 0.006 mg (Quercetin equivalents) per g of extracts (standard curve equation: *y* = 0.0040*x* + 0.0085, *R* ^2^ = 0.991) and a ratio flavonoids/phenolics of 0.58. Thus, phenolic compounds were the predominant antioxidant components in *O. stamineus *extracts, which lead to more potent radical scavenging effect.

### 3.3. Histopathology Examination

**Figure 1:**
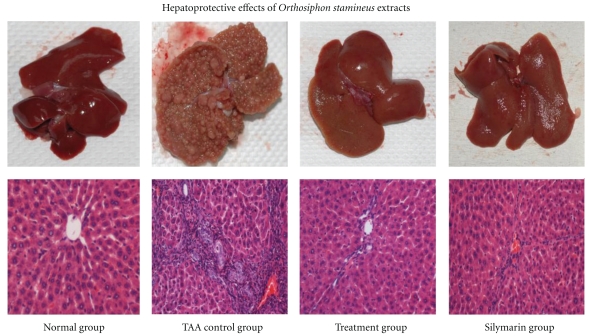
Effect of TAA, Silymarin, and 200 mg/kg *O. stamineus *ethanolic extract on liver gross and histology in TAA-Induced liver cirrhosis rats after two-month treatments. Eight animals of each group were investigated.

Histopathological examination of liver sections of the normal group showed regular cellular architecture with distinct hepatic cells, sinusoidal spaces, and a central vein. The hepatocytes are polygonal cells with well preserved cytoplasm, nucleus with prominent nuclei. On the other hand, in the hepatotoxic positive control group, histological examination showed loss of architecture, inflammation, and congestion with cytoplasmic vacuolation, fatty change, sinusoidal dilatation, centrilobular necrosis, and displayed bundles of collagen surrounding the lobules, which resulted in huge fibrous septa and distorted tissue architecture. In *O. stamineus*-treated animals, liver sections showed mild inflammation and mild necrosis of hepatocytes with mild cytoplasmic vacuolation, and mostly no visible changes observed. Histopathological examination also showed good recovery of thioacetamide-induced necrosis by ethanolic extracts as compared to Silymarin. Animals treated with the low dose showed regeneration of hepatocytes surrounded by septa of fibrous tissue with a significant increase in bile ductules, fat storing cells, and Kupffer cells. Animals treated with the higher dose of plant extract showed remarkable histological regeneration compared to those of the LD group. They showed nearly ordinary patterns with an increase normal hepatocytes parenchyma and a reduced development of fibrous septa and lymphocyte infiltration. Results of the gross and histopathological examination are shown in the figures (Figure 1).

## 4. Discussion

Toxic injury occurs in the liver more often than that in any other organ. When a drug is used widely, drug-induced liver injury has become a serious health problem in contemporary society, then research on the mechanism of drug-induced liver injury is very useful in therapy and prevention of drug-induced liver injury [22]. Thioacetamide is known hepatotoxic, which produces hepatic necrosis in high doses by producing free radicals during TAA metabolism resulting in oxidative stress mediated acute hepatitis and induces apoptosis of hepatocytes in the liver [23]. It has been reported that long-term taken of TAA induced cirrhosis in rats; on account of this, it is proven that thioacetamide through cytochrome p-450 pathway is converted into a highly toxic metabolite N-acetyl-p-benzoquinone imine (NAPBI). Meanwhile, (NAPBI) is accompanied with glutathione and excreted in the urine as conjugates. The acute hepatic necrosis induced by TAA, which activates cytochrome p450 and produces a highly reactive NAPBQI that, by the way, combines with sulpha-hydryl groups of proteins and causes a rapid reduction of intracellular glutathione. Therefore, increases the oxygen free radical causing an oxidative stress and initiates apoptosis; consequently, the elevated liver enzymes (ALT, AST) are an indicator of cellular liver necrosis [24]. In addition, TAA interferes with the movement of RNA from the nucleus to the cytoplasm which may cause membrane injury resulting in a rise in serum liver markers [25]. TAA toxic metabolite free radicals induced oxidative stress in the hepatic cells. It is responsible for many changes occur for hepatocytes such as an increase in nuclear volume and enlargement of nucleoli, cell permeability changes, rise in intracellular concentration of Ca++, and effects on mitochondrial activity, which leads to cell death [19].

Liver damage is associated with cellular necrosis, increases in tissue lipid peroxidation as MDA and level caused by oxidative stress and depletion in the tissue GSH levels. Moreover, serum levels of liver function parameters like ALT, AST, bilirubin, and alkaline phosphatase are elevated. The mechanism of liver fibrosis is not understood, but no doubt that oxidative stress and reactive oxygen species (ROS) play an important role in pathological changes in the liver. In this study, TAA administration for eight weeks led to induced liver fibrosis, which has been proven by the significantly difference of biochemical markers between the TAA control and normal control groups. At the same time, the hepatoprotective effect exhibited by *O. stamineus *at dose 200 mg/kg was comparable to Silymarin at dose 50 mg/kg in TAA-induced liver injury rats. Treatment with the ethanolic extracts of *O. stamineus *leaves (200 mg/kg) has accelerated the return of the altered levels of liver function enzyme and to the near normal profile. The abnormal reconstruction of the lobular architecture, the appearance of widespread fibrosis in addition, nodular lesions of the hepatic parenchyma are the main characteristics of liver cirrhosis [26]. Our histological findings prove that the ethanol extracts of *O. stamineus *affected the recovery of liver structure in TAA-induced liver cirrhosis rats. Indeed, there was remarkable reduction in fibrosis extent and a decrease of stellate infiltration in rats treated with plant extract compared to control TAA group. Histological studies confirmed the hepatoprotective effect of *O. stamineus* ethanolic extract. TAA treated rat liver sections showed fatty degeneration of hepatocytes and necrosis of cells. The extract treatment (200 mg/kg) almost normalized these effects in the histoarchitecture of liver. Furthermore, the severe fatty changes in the livers of rats caused by TAA were treated in the HD treatment groups. Therefore, from this study the ethanol extracts of *O. stamineus* could be a hepatoprotective against thioacetamide induced liver damage in rats.

The antioxidant capabilities of the phenolic compounds are important for the human body to destroy the free radicals that exist in our body. Many of the polyphenols such as flavonoids have been identified as powerful antioxidants; moreover, play a significant role in the treatment of many diseases, including liver cirrhosis [27]. On the other hand, there was a study on the effect of *Silybum marianum *and *Cichorium intybus *extracts on liver cells suggested that hepatoprotective action due to the presence of flavonoids and their antioxidant effects [28]. *O. stamineus *has been reported to possess antioxidant activity; furthermore, the extracts exhibited significant radical-scavenging activity probably due to the higher concentration of caffeic acid derivatives, especially rosmarinic acid [9]. By the way, Akowuah also found that the *O. stamineus *extract antioxidative potency was higher than a synthetic antioxidant butylated hydroxylanisole (BHA) and almost equal to that of pure quercetin [29]. Similarly, the extract show increase in activities of antioxidant enzymes such as CAT and SOD [30]. In this study, reduced lipid peroxidation was revealed by a significant decrease in MDA level in groups treated with ethanol extracts. The results of the hepatoprotective effects of this extracts can be due to the presence of the great amount of phenolic and flavonoids compounds and their antioxidant effects besides the free radical scavenging property of this plant. Likewise, the hepatoprotective activity of the extract could be due to neutralization of the toxic compounds produced by converting TAA to a highly toxic metabolite during cytochrome p-450 pathway as mentioned above. On account of this *O. stamineus* extract, it has been reported recently to affect cytochrome p450 enzyme system through its inhibition. Consequently, the toxic metabolite of TAA is affected by the *O. stamineus* extract that might lead to reduce the progress of liver necrosis [31]. 

In conclusion, this study showed that *O. stamineus *ethanol extracts have hepatoprotective effects that were proven by biochemical and histopathological analysis. Accordingly, the plant extracts could be an effective herbal for chemical-induced hepatic damage although this finding needs further study to know the active constituents appearing to protect rat liver against cirrhosis.

## Figures and Tables

**Table 1:** The body, liver, and spleen weight of rats after two-month treatments.

Animal group	Body weight (g) (BW)	Liver weight (g) (LW)	LW/BW (%)	Spleen weight (g) (SW)	SW/BW (%)
Normal control	254.9 ± 28.69	6.71 ± 0.64	2.71 ± 0.18	0.47 ± 0.08	0.18 ± 0.02
TAA control (hepatotoxic group)	202.0 ± 19.10	11.00 ± 1.11^∗a^	5.43 ± 0.17^∗a^	0.52 ± 0.07	0.26 ± 0.03
HD 200 mg/kg (treatment group)	232.7 ± 16.12	10.43 ± 0.69	4.50 ± 0.19^∗b^	0.54 ± 0.04	0.23 ± 0.01
LD 100 mg/kg (treatment group)	263.3 ± 8.53	10.43 ± 0.72	3.94 ± 0.16^∗∗c^	0.55 ± 0.03	0.21 ± 0.01
Silymarin 50 mg/kg (hepatoprotective group)	257.0 ± 21.97	7.71 ± 2.78	2.94 ± 0.13^∗∗c^	0.53 ± 0.07	0.20 ± 0.01

All values are expressed as mean ± S.E.M. Means with different superscripts are significantly different.

^a^ *P* < .05 versus Normal control group, ^b^ *P* < .05 versus TAA control group, and ^c^ *P* < .01 versus TAA control group.

**Table 2:** Effect of TAA, Silymarin, and *O. stamineus *ethanolic extract on biochemical parameters in thioacetamide-Induced liver cirrhosis rats.

Animal group	ALT (IU/L)	AST (IU/L)	ALP (IU/L)	Bilirubin (mg/dl)	T.Protein (g/l)	Albumin (g/l)	MDA nmol/mL
Normal control	64.9 ± 4.19^a^	164.4 ± 10.74^a^	109.6 ± 9.80^a^	1.86 ± 0.1^a^	74.3 ± 1.15^a^	12.1 ± 0.51^a^	38.7 ± 2.6^a^
TAA Control	213.3 ± 25.98^d^	372.6 ± 29.98^d^	435.8 ± 29.78^d^	8.7 ± 0.57^d^	60.7 ± 0.97^d^	8.3 ± 0.57^d^	107.1 ± 3.7^d^
HD 200 mg/kg	95.7 ± 9.35^b^	228.6 ± 14.10^b^	289.0 ± 14.23^c^	4.8 ± 0.59^b^	68.0 ± 2.06^c^	11.1 ± 0.63^c^	45.3 ± 3.5^b^
LD 100 mg/kg	108.0 ± 11.15^c^	253.4 ± 18.67^c^	383.6 ± 20.89	6.4 ± 0.72^c^	64.6 ± 1.29	9.3 ± 0.36	72.6 ± 3.9^c^
Silymarin 50 mg/kg	70.4 ± 5.60^b^	171.6 ± 10.19^b^	139.4 ± 9.54^b^	3.0 ± 0.31^b^	70.9 ± 0.91^b^	11.7 ± 0.68^b^	40.3 ± 2.8^b^

All values are expressed as mean ± S.E.M. of eight rats in each group. Values not sharing a common superscript differ significantly, *P < .05. ALT: alanine aminotransferase, AST: aspartate aminotransferase, ALP: alkaline phosphatase, and MDA: malondialdehyde.
